# Correspondence: Reply to ‘On the nature of strong piezoelectricity in graphene on SiO_2_'

**DOI:** 10.1038/ncomms11571

**Published:** 2016-05-17

**Authors:** Gonçalo da Cunha Rodrigues, Pavel Zelenovskiy, Konstantin Romanyuk, Sergey Luchkin, Yakov Kopelevich, Andrei Kholkin

**Affiliations:** 1 Department of Physics & CICECO—Aveiro Institute of Materials, University of Aveiro, 3810-193 Aveiro, Portugal; 2 Institute of Natural Sciences, Ural Federal University, Ekaterinburg 620000, Russia; 3 Instituto de Física, UNICAMP, Campinas, São Paulo 13083-859, Brasil

In our paper^1^ we provided an experimental evidence that the single-layer graphene (SLG) deposited on SiO_2_ grating substrate exhibits very strong out-of-plane piezoelectric effect, several times greater than that of the best piezoceramics such as lead-zirconate titanate. Simultaneously, the in-plane strain distribution was measured by micro-Raman scattering in an attempt to relate such unusual activity to the strain gradient expected in suspended graphene near the ridges. We appreciate the comment by Stampfer and Reichardt^2^ who noticed that our calculations of the in-plane strain based on ref. 3 were incorrect. The overestimation of the strain values, however, does not change the main conclusion of our paper, since the piezoresponse force microscopy measurements give the value of out-of-plane a.c. deformation, fully decoupled from the d.c. in-plane strain measured by micro-Raman. We recalculated the strain map and corrected the strain values that vary now in the range from −0.078 to +0.078%. Nevertheless, the strain ratio between supported and suspended graphene is still 2.5 (strain for suspended graphene is about +0.02%). Figure 1a,b displays both the corrected strain map and cross-section of the position of Raman line and recalculated strain. Below we reply in detail to other issues raised by Stampfer and Reichardt in their communication^2^.

Gruneisen parameter suggested by Stampfer and Reichardt^2^ is related to graphene produced by mechanical exfoliation, whereas in our work^1^ we used graphene sheets prepared by CVD. These two materials differ by the number of intrinsic defects and, therefore, their behaviour under uniaxial or biaxial strain is different. This was exactly demonstrated in ref. 3, where the opposite sign of Gruneisen parameter for G-band was revealed for CVD graphene with respect to the exfoliated one. Therefore, we used the value reported in ref. 3 for our calculations which gave us a maximum strain of 0.078%.

Stampfer and Reichardt^2^ questioned why we used the Gruneisen parameter for uniaxial strain. We believe that using the value for uniaxial strain is dictated by the nature of the substrate. Being periodical in one direction only, this feature naturally defines the symmetry of the graphene deformations as well. Therefore, we considered uniaxial strain in our work.

Stampfer and Reichardt^2^ claim that our paper ‘…directly linked the observed piezoelectricity in graphene to the supposed high in-plane strain induced by the substrate…'. Indeed, the observed piezoresponse distribution (Fig. 3(b) of ref. 1) apparently correlates with the mechanical strain distribution in graphene. However, it does not mean that the observed piezoelectricity is associated with this strain (and its value!). Since no amplification of the piezoresponse is observed near the grating edges where the maximum strain gradient is expected, we only ruled out the in-plane symmetry breaking induced by discussed in-plane strain. Therefore, the conclusion of the paper remains the same, that is, chemical bonds generated between O and C atoms can indeed induce piezoelectric effect and, at the same time, affect the G-band position.

Stampfer and Reichardt^2^ cast some doubt that the C–O bonds can form at the interface with graphene based on some data on carrier mobility. The formation of strong C–O bonds between graphene and SiO_2_ has been already proven both experimentally and theoretically (see, for example, refs 3, 4, 5, 6). This fact is strongly supported by the observed p-type conductivity in graphene via a charge transfer from the carbon in graphene layer to the oxygen-terminated surface of SiO_2_ (ref. 4). As such, the observation of a strong piezoelectricity due to formation of polar C–O bonds is not a surprise. Though some authors^7^ claim that only weak bonds are formed for the most stable geometry of C atoms, the very existence of the strong polarity of the graphene–SiO_2_ interface proves that it is not the case.

All in all, we highly appreciate the comment by Stampfer and Reichard^1^ concerning the overestimation of the in-plane strain, however, it does not change the main conclusion of the paper, that is, the experimental observation of strong piezoelectricity in graphene/SiO_2_. We emphasize that, while an inhomogeneous interaction between graphene and underlying SiO_2_ substrate (caused for example, by the substrate morphology) can be responsible for the in-plane strain seen in Raman measurements, the observed piezoresponse is attributed to the formation of out-of-plane polar C–O bonds that may not be directly related to the in-plane deformation.

## Additional information

**How to cite this article:** da Cunha Rodrigues, G. *et al*. Correspondence: Reply to ‘On the nature of strong piezoelectricity in graphene on SiO_2_'. *Nat. Commun.* 7:11571 doi: 10.1038/ncomms11571 (2016).

## Figures and Tables

**Figure 1:**
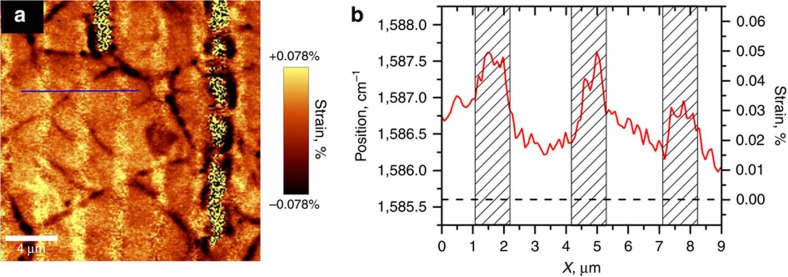
Strains in the single-layer graphene on the silicon grating. (**a**) Strain map. (**b**) Variation of G-band position and strain across the grating (blue line in **a**); shaded rectangles correspond to supported graphene, dashed line denotes the initial (unstressed) value of G-band position.
